# Biofilm Adherence and Detachment Pathway Elucidated

**DOI:** 10.1371/journal.pbio.1001012

**Published:** 2011-02-01

**Authors:** Richard Robinson

**Affiliations:** Freelance Science Writer, Sherborn, Massachusetts, United States of America

**Figure:**
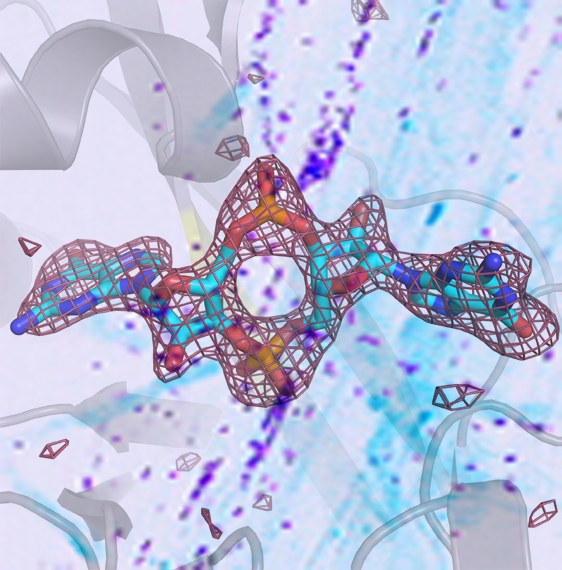
The structure of cyclic dimeric-GMP (c-di-GMP) bound to the receptor LapD from *P. fluorescens* is depicted. The background shows an image of *P. fluorescens* colonizing a plant root.

Dental plaque and the slime around the shower drain are both biofilms, as is the slippery coating often found on river rocks, or inside an overused and underwashed water bottle. Biofilms form when bacteria give up their free-swimming ways and adhere to a surface; their secretions give the film its slimy texture. For ill or good (biofilms also play a part in sewage treatment, for instance), biofilms are ubiquitous. They are also tenacious—eradication of a biofilm, or controlling its growth once it forms, can be a significant challenge.

But biofilms can and do disperse on their own, especially in response to nutrient deprivation in the environment, including low phosphate. Bacteria need phosphate, and when it is in short supply, it makes little sense to stay put—better to pull up stakes and float away to greener pastures. For selected bacteria, adhesion is largely the job of a single protein. For the model organism *Pseudomonas fluorescens*, the adhesin is called LapA (large adhesion protein A). With LapA in the outer membrane, bacteria stick; when it is lost from the membrane, they don't.

The molecular signaling pathway that controls LapA, and thus adhesion and detachment, has recently begun to be understood. Two new papers in this issue add greatly to that understanding, providing a detailed outline of the regulatory mechanism and the structure of the key molecule involved.

Peter Newell, George O'Toole, and colleagues examined the details of the phosphate-induced signaling pathway of *P. fluorescens*. High phosphate in the environment supports the production within the bacterium of cyclic dimeric-GMP (c-di-GMP), a bacterium-specific second messenger that binds to a diverse group of receptors, or effector molecules. Their group previously characterized one effector, known as LapD, and showed it acted upstream of LapA to control adhesion. Here, they began by focusing on a gene adjacent to the LapD gene, called LapG.

Deletion of LapG promoted biofilm formation, coinciding with an accumulation of LapA on the surface of the bacterium, suggesting LapG promoted loss of LapA from the membrane and detachment of cells from the substratum. They showed that LapG was a protease that could cleave LapA, and found that LapG resided in the periplasmic space (i.e., between the two bacterial membranes), where it would have access to LapA in the outer membrane.

LapD's effects were opposite those of LapG—deletion of LapD reduced biofilm formation, while overexpression promoted LapA's retention on the outer membrane. LapD and LapG precipitated together, indicating they directly interact, and depletion of c-di-GMP reduced their interaction.

Meanwhile, Marcos Navarro, Holger Sondermann, and colleagues (Sondermann and O'Toole worked together on both studies) examined the molecular structure of LapD both with and without c-di-GMP.

LapD, which is embedded in the inner bacterial membrane, has several distinct domains, including a V-shaped periplasmic domain, and an elaborate set of cytoplasmic modules, including the c-di-GMP binding pocket.

They found that the presence of c-di-GMP in the pocket exposed an otherwise hidden face of the protein. That face on one molecule of LapD bound to the same face on another, linking them together. Binding of c-di-GMP and that linkage caused a conformation change in the entire protein that was transmitted through the membrane to the V-shaped periplasmic domain. When the V adopted a different state, it made room for binding LapG. Conversely, absence of c-di-GMP hid the linking faces of the two LapDs, shifting the V to a closed position, preventing binding of LapG.

Taken together, the results of the two studies suggest a model for regulation of phosphate-dependent cell adherence. When phosphate is high, c-di-GMP binds to LapD, likely altering the V shape and sequestering LapG. With LapG bound up and LapA in the outer membrane, the bacterium adheres and the biofilm grows. As phosphate becomes depleted, c-di-GMP is lost from LapD. The V again alters shape, LapG is released to cleave LapA, and the bacterium detaches from the substrate, free to wander off in search of new phosphate.

Given the economic and health importance of biofilms, the full understanding of this regulatory system is likely to have significant and immediate practical applications. Blocking the binding of LapD and LapG, for instance, could help clear marine pipelines lined with bacterial films that clog and corrode the pipes. Stubborn and deadly biofilms can adhere to heart valves, artificial joints, and other implanted medical devices. Finding ways to prevent their accumulation or disperse them after they have formed may save lives. Other “inside-out” signaling pathways in other bacteria are likely regulated through a similar mechanism, suggesting the mechanistic insights from these studies will be applicable beyond biofilms, as well.

**Newell PD, Boyd CD, Sondermann H, O'Toole GA (2011) A C-di-GMP Effector System Controls Cell Adhesion by Inside-out Signaling and Surface Protein Cleavage. doi:10.1371/journal.pbio.1000587**

**Navarro MVAS, Newell PD, Krasteva PV, Chatterjee D, Madden DR, et al. (2011) Structural Basis for c-di-GMP-mediated Inside-out Signaling Controlling Periplasmic Proteolysis. doi:10.1371/journal.pbio.1000588**

